# Supplementary material for: Risk of SARS-CoV-2 reinfection during multiple Omicron variant waves in the UK general population
Source: Nat Commun. 2024 Feb 2;15:1008. doi: 10.1038/s41467-024-44973-1 (PMC10837445; doi:10.1038/s41467-024-44973-1)
Supplement: Supplementary file 1 — Supplementary Information [file 41467_2024_44973_MOESM1_ESM.pdf]

Supplementary information for

Risk of SARS-CoV-2 reinfection during multiple Omicron variant waves in the UK general population

Jia Wei, Nicole Stoesser, Philippa C. Matthews, Tarnjit Khera, Owen Gethings, Ian Diamond, Ruth Studley, Nick Taylor, Tim E. A. Peto, A. Sarah Walker, Koen B. Pouwels, David W. Eyre, and the COVID-19 Infection Survey team

Contents

- **Supplementary Tables 1-5**
- **Supplementary Figures 1-12**

				First infection			Second infection			Third infection			Fourth infection			Fifth infection		
	**Age group at first infection**	**Sex**	**Ethnicity**	Variant	Symptom	Ct value/LFD positive	Variant	Symptom	Ct value/LFD positive	Variant	Symptom	Ct value/LFD positive	Variant	Symptom	Ct value/LFD positive	Variant	Symptom	Ct value/LFD positive
**1**	**46-50**	**Female**	**White**	Pre-Alpha	Classic	32	Delta	Classic	Not positive	Delta	Yes	Not positive	BA.2	Classic	Positive	BA.4/5	No	Positive
**2**	**46-50**	**Female**	**White**	AY.4*	Classic	Not positive	BA.1	Yes	Not positive	BA.2	Classic	Positive	BA.4/5	Classic	Not positive	BA.5.1*	Classic	Positive
**3**	**41-45**	**Female**	**White**	Delta	Yes	Positive	BA.2	No	Positive	BA.4/5	No	Positive	BA.4/5	No	Positive	CA.7*	Classic	Not positive
**4**	**21-25**	**Female**	**White**	Delta	Yes	Not positive	BA.1	No	Positive	BA.2	Classic	Not positive	BF.27*	No	Positive	BQ.1/CH.1.1/XBB.1.5	Classic	Not positive
**5**	**36-40**	**Female**	**White**	Pre-Alpha	Classic	29	Delta	No	28	Delta	Yes	Positive	BA.1*	Yes	18	BA.4/5	No	Positive
**6**	**36-40**	**Female**	**White**	Pre-Alpha	Classic	Not positive	BA.1.1*	Classic	18	BA.2	Classic	Positive	BQ.1/CH.1.1/XBB.1.5	Classic	Positive	BE.9*	Classic	16
**7**	**41-45**	**Female**	**White**	Delta	Yes	Not positive	BA.1	Yes	21	BA.2	Yes	Not positive	BA.4/5	Classic	Not positive	BQ.1.1*	Classic	28

Supplementary Table 1. Characteristics of infections in 7 participants who had five infections. Age was categorised into 5-year groups for anonymisation. We used pangolin sequence assignment if available (marked as *), otherwise defined the infection variant based on calendar time (see Methods). The Ct value associated with each infection was the minimum observed across all positive tests within each infection (see Methods). If no Ct was recorded, we classified the infection by whether there were any lateral flow device (LFD) positive results from the national testing programmes in the infection. Symptoms were classified as 'yes' or 'no' for all participants. If specific symptoms were reported (only available from survey questionnaires, not available from national testing programmes), they were further classified as 'classic' (any of cough, fever, loss of taste/smell) or 'other' (myalgia, fatigue/weakness, sore throat, shortness of breath, headache, diarrhoea, nausea, abdominal pain).

	Model including first, second, and third infections				Model including only second and third infections			
	Difference in Ct value in current infection	95%CI		p value	Difference in Ct value in current infection	95%CI		p value
Age (per 10-year older)	-0.041	-0.059	-0.023	**1.1e-05**	0.082	0.020	0.144	**0.01**
Sex (Male vs Female)	-0.456	-0.510	-0.403	**<2.2e-16**	-0.481	-0.648	-0.315	**1.4e-08**
Ethnicity (Non-white vs White)	-0.101	-0.207	0.004	0.06	-0.033	-0.340	0.273	0.8
Working in healthcare (Yes vs No)	-0.195	-0.317	-0.074	**0.002**	-0.196	-0.554	0.161	0.3
Having a long-term health condition (Yes vs No)	0.044	-0.020	0.108	0.2	-0.188	-0.390	0.013	0.07
Deprivation percentile (per 10 percentile higher)	0.031	0.021	0.041	**8.0e-10**	0.001	-0.029	0.031	0.9
Reported symptoms in this infection (Yes vs No)	-3.639	-3.704	-3.573	**<2.2e-16**	-2.571	-2.756	-2.385	**<2.2e-16**
Reported symptoms in previous infection (Yes vs No)					0.169	0.002	0.380	**0.04**
Number of positive tests in the infection (per one more)	-0.109	-0.137	-0.081	**2.8e-14**	0.341	0.201	0.485	**3.9e-06**
Variant (Pre-Alpha vs Omicron BA.1)	4.568	2.536	6.600	**1.1e-05**	5.766	0.205	11.237	**0.04**
Variant (Alpha vs Omicron BA.1)	1.555	-2.032	5.141	0.4	5.575	3.939	7.211	**2.5e-11**
Variant (Delta vs Omicron BA.1)	-0.774	-1.017	-0.531	**4.0e-10**	-0.113	-1.446	1.220	0.9
Variant (Omicron BA.2 vs Omicron BA.1)	1.586	1.370	1.801	**<2.2e-16**	2.426	1.781	3.070	**1.7e-13**
Variant (Omicron BA.4/5 vs Omicron BA.1)	2.164	1.977	2.350	**<2.2e-16**	2.175	1.632	2.719	**4.7e-15**
Variant (Omicron BQ.1/CH.1.1/XBB.1.5 vs Omicron BA.1)	3.116	2.892	3.341	**<2.2e-16**	3.090	2.541	3.640	**<2.2e-16**
Time from most recent vaccination (No vaccination vs >180 days)	-0.125	-0.540	0.290	0.6	-0.166	-1.164	0.832	0.8
Time from most recent vaccination (14-90 days vs >180 days)	0.184	0.002	0.366	**0.04**	1.065	0.467	1.662	**4.8e-04**
Time from most recent vaccination (90-180 days vs >180 days)	0.429	0.235	0.623	**1.5e-05**	1.033	0.370	1.696	**0.002**
Infection (2 vs 1)	0.810	0.554	1.065	**5.0e-10**				
Infection (3 vs 1)	2.378	1.050	3.707	**4.5e-04**	0.342	0.006	0.677	**0.04**
Variant: Time from most recent vaccination (interaction)				**<2.2e-16**				**1.1e-08**
Pre-Alpha:No vaccination	-2.732	-4.797	-0.667	**0.01**	-1.488	-7.613	4.638	0.6
Alpha:No vaccination	0.222	-3.384	3.828	0.8	-1.537	-3.763	0.689	0.2
Delta:No vaccination	0.173	-0.360	0.706	0.5	1.179	-1.086	3.444	0.3
Omicron BA.2:No vaccination	-0.586	-1.226	0.054	0.07	-1.496	-2.953	-0.039	**0.04**

	Model including first, second, and third infections				Model including only second and third infections			
	Difference in Ct value in current infection	95%CI		p value	Difference in Ct value in current infection	95%CI		p value
**Omicron BA.4/5:No vaccination**	0.565	-0.220	1.349	0.2	0.715	-0.779	2.209	0.4
**Omicron BQ.1/CH.1.1/XBB.1.5:No vaccination**	0.034	-0.830	0.897	0.9	0.050	-1.495	1.594	0.9
**Alpha:14-90 days**	1.752	-1.853	5.357	0.3				
**Delta:14-90 days**	0.233	-0.053	0.519	0.1	2.037	0.442	3.651	**0.01**
**Omicron BA.2:14-90 days**	-0.860	-1.124	-0.596	**1.6e-10**	-1.712	-2.555	-0.869	**6.8e-05**
**Omicron BA.4/5:14-90 days**	-0.100	-0.394	0.194	0.5	-0.303	-1.186	0.579	0.5
**Omicron BQ.1/CH.1.1/XBB.1.5:14-90 days**	-0.251	-0.518	0.016	0.07	-0.989	-1.674	-0.303	**0.005**
**Delta:90-180 days**	-0.404	-0.685	-0.123	**0.005**	-1.270	-2.842	0.302	0.1
**Omicron BA.2:90-180 days**	-0.945	-1.190	-0.701	**3.5e-14**	-1.828	-2.636	-1.019	**9.5e-06**
**Omicron BA.4/5:90-180 days**	-0.903	-1.191	-0.615	**7.8e-10**	-1.528	-2.396	-0.660	**5.6e-04**
**Omicron BQ.1/CH.1.1/XBB.1.5:90-180 days**	-0.655	-0.936	-0.374	**4.9e-06**	-1.240	-1.991	-0.490	**0.001**
**Variant:Infection (interaction)**				**1.6e-08**				
**Pre-Alpha:infection 2**	2.248	0.414	4.081	**0.02**				
**Alpha:infection 2**	1.878	0.939	2.818	**8.9e-05**				
**Delta:infection 2**	0.751	0.292	1.211	**0.001**				
**Omicron BA.2:infection 2**	0.051	-0.217	0.319	0.7				
**Omicron BA.4/5:infection 2**	-0.227	-0.510	0.056	0.1				
**Omicron BQ.1/CH.1.1/XBB.1.5:infection 2**	-0.366	-0.621	-0.112	**0.005**				
**Delta:infection 3**	-2.105	-6.547	2.363	0.4				
**Omicron BA.2:infection 3**	-1.373	-2.869	0.122	0.07				
**Omicron BA.4/5:infection 3**	-1.482	-2.871	-0.093	**0.04**				
**Omicron BQ.1/CH.1.1/XBB.1.5:infection 3**	-1.737	-3.045	-0.430	**0.009**				
**Time from most recent vaccination: Infection**				**3.4e-06**				
**No vaccination:infection 2**	-0.540	-1.066	-0.015	**0.04**				
**14-90 days:infection 2**	0.483	0.249	0.717	**5.2e-05**				
**90-180 days:infection 2**	-0.024	-0.247	0.199	0.8				

	Model including first, second, and third infections				Model including only second and third infections			
	Difference in Ct value in current infection	95%CI		p value	Difference in Ct value in current infection	95%CI		p value
**No vaccination:infection 3**	-1.758	-3.226	-0.180	**0.03**				
**14-90 days:infection 3**	-0.328	-1.056	0.400	0.4				
**90-180 days:infection 3**	-0.108	-0.812	0.596	0.8				

Supplementary Table 2. Effect estimates and 95% confidence intervals (95%CIs) in a normal linear regression model examining the association between Ct values and different characteristics. n=185,484 infections were included in the model. Two-sided t-test was used to test the significance of model coefficients. The 95% CIs are calculated as estimates ± 1.96 × standard error of the estimates. Combined effects from interactions between variant, time from the most recent vaccination, and infection number are also shown in **Fig. 1.** A separate model was built only including reinfections (second and third infections) and specifically examined the effects of Ct values and reported symptoms in the most recent previous infection (effects from Ct values are shown in **Supplementary Fig. 3**).

(A)	Reinfection in Omicron BA.1 wave (27 December 2021 to 6 February 2022)				Reinfection in Omicron BA.2 wave (14 March 2022 to 22 May 2022)				Reinfection in Omicron BA.4/5 wave (27 June 2022 to 6 November 2022)				Reinfection in Omicron BQ.1/CH.1.1/XBB.1.5 subvariants wave (7 November 2022 to 31 January 2023)			
	HR	95%CI		p-value	HR	95%CI		p-value	HR	95%CI		p-value	HR	95%CI		p-value
**Sex (Male vs Female)**	0.91	0.83	0.99	**0.03**	0.85	0.79	0.91	**8.6e-06**	0.87	0.83	0.90	**4.5e-12**	0.91	0.87	0.95	**1.6e-06**
**Ethnicity (Non-white vs White)**	0.92	0.79	1.06	0.2	0.71	0.61	0.81	**1.9e-06**	0.88	0.82	0.95	**0.002**	0.82	0.76	0.89	**1.6e-06**
**Healthcare worker (Yes vs No)**	1.26	1.08	1.48	**0.003**	1.10	0.95	1.28	0.2	1.05	0.96	1.13	0.3	1.17	1.08	1.26	**1.3e-04**
**Long-term health condition (Yes vs No)**	0.95	0.85	1.06	0.4	1.02	0.94	1.11	0.6	1.06	1.01	1.12	**0.01**	1.07	1.02	1.12	**0.003**
**Deprivation percentile**	0.98	0.97	1.00	**0.04**	1.01	1.00	1.03	**0.03**	1.00	0.99	1.00	0.4	0.99	0.99	1.00	0.1
**Symptom in most recent previous infection (Yes vs No)**	0.96	0.86	1.08	0.6	0.92	0.84	1.01	0.08	1.04	0.98	1.10	0.2	1.09	1.03	1.14	**0.001**
**Any Ct<30 or LFD positive in all previous infections**	1.00	0.79	1.26	0.9	0.81	0.67	0.97	**0.03**	1.01	0.92	1.12	0.8	1.08	0.99	1.19	0.09
**Region (Northern Ireland vs England)**	0.58	0.39	0.85	**0.005**	0.64	0.45	0.91	**0.01**	0.94	0.82	1.08	0.4	1.10	0.98	1.24	0.1
**Region (Scotland vs England)**	0.75	0.56	0.98	**0.04**	0.77	0.64	0.93	**0.006**	0.98	0.89	1.07	0.6	0.99	0.91	1.08	0.7
**Region (Wales vs England)**	0.43	0.27	0.69	**3.8e-04**	0.86	0.70	1.06	0.2	0.96	0.86	1.07	0.4	1.04	0.95	1.15	0.4
**Number of previous infections (2 vs 1)**	0.93	0.64	1.37	0.7	1.06	0.83	1.35	0.6	1.03	0.94	1.11	0.6	1.01	0.94	1.08	0.7
**Number of previous infections (3 vs 1)**									1.16	0.70	1.93	0.6	1.39	1.02	1.89	**0.04**
**Per 60 days after a most recent Pre-Alpha infection**	1.00	0.89	1.13	0.9	0.98	0.87	1.11	0.8	0.94	0.80	1.10	0.4	0.88	0.63	1.22	0.4
**Per 60 days after a most recent Alpha infection**	0.95	0.85	1.06	0.4	1.00	0.90	1.11	0.9	0.91	0.84	0.98	**0.009**	0.85	0.76	0.95	**0.006**
**Per 60 days after a most recent Delta infection**	0.77	0.63	0.93	**0.008**	1.14	1.07	1.21	**4.2e-05**	1.00	0.97	1.04	0.9	0.89	0.85	0.93	**1.2e-07**
**Per 60 days after a most recent Omicron BA.1 infection**									1.18	1.12	1.24	**2.1e-11**	0.96	0.91	1.01	0.09
**Per 60 days after a most recent Omicron BA.2 infection**									1.32	1.19	1.45	**8.0e-08**	1.19	1.14	1.25	**1.7e-12**
**Per 60 days after a most recent Omicron BA.4/5 infection**													1.46	1.30	1.64	**3.7e-10**
**Prevalence (per 1% higher)**	1.20	1.14	1.26	**2.0e-12**	1.23	1.16	1.31	**4.7e-12**	1.33	1.26	1.41	**<2e-16**	1.31	1.25	1.37	**<2e-16**

**(B)**	**HR (95% CI)**	**Pre-alpha**	**Alpha**	**Delta**
**Pre-alpha**	1.00 (0.89-1.13)	/	P=0.6	**P=0.01**
**Alpha**	0.95 (0.85-1.06)	/	/	**P=0.03**
**Delta**	0.77 (0.63-0.93)	/	/	/

**(C)**	**HR (95% CI)**	**Pre-alpha**	**Alpha**	**Delta**
**Pre-alpha**	0.98 (0.87-1.11)	/	P=0.8	**P=0.03**
**Alpha**	1.00 (0.90-1.11)	/	/	**P=0.03**
**Delta**	1.14 (1.07-1.21)	/	/	/

(D)	HR (95% CI)	Pre-alpha	Alpha	Delta	BA.1	BA.2
**Pre-alpha**	0.94 (0.80-1.10)	/	P=0.7	P=0.4	**P=0.006**	**P=0.0003**
**Alpha**	0.91 (0.84-0.98)	/	/	**P=0.02**	**P=7.8e-09**	**P=5.7e-09**
**Delta**	1.00 (0.97-1.04)	/	/	/	**P=1.4e-07**	**P=5.8e-07**
**BA.1**	1.18 (1.12-1.24)	/	/	/	/	P=0.05
**BA.2**	1.32 (1.19-1.45)	/	/	/	/	/

(E)	HR (95% CI)	Pre-alpha	Alpha	Delta	BA.1	BA.2	BA.4/5
**Pre-alpha**	0.88 (0.63-1.22)	/	P=0.9	P=0.9	P=0.6	P=0.08	**P=0.006**
**Alpha**	0.85 (0.76-0.95)	/	/	P=0.5	P=0.1	**P=9.9e-07**	**P=1.8e-09**
**Delta**	0.89 (0.85-0.93)	/	/	/	**P=0.04**	**P=2.2e-16**	**P=3.8e-13**
**BA.1**	0.96 (0.91-1.01)	/	/	/	/	**P=1.6e-08**	**P=1.4e-09**
**BA.2**	1.19 (1.14-1.25)	/	/	/	/	/	**P=0.003**
**BA.4/5**	1.46 (1.30-1.64)	/	/	/	/	/	/

Supplementary Table 3. (A) Adjusted hazard ratios (HRs) with 95% CIs from parametric survival models examining the risk of reinfection in multiple Omicron infection waves (Omicron BA.1, BA.2, BA.4/5, and BQ.1/CH.1.1/XBB.1.5). n=42,582, 83,382, 164,263, and 184,566 adults who were at risk of BA.1, BA.2, BA.4/5, and BQ.1/CH.1.1/XBB.1.5 reinfections were included in the models, respectively. Two-sided z-test was used to test the significance of model coefficients. The 95% CIs are calculated by the exponent of the estimates ± 1.96 × standard error of the estimates. Effects of time from previous infection are shown in **Fig. 3,** from time from most recent vaccination in **Fig. 4,** and of age and Ct values in **Supplementary Figs. 5, 6**. Effects from other covariates are shown in **Fig. 2**. Results remain broadly similar in sensitivity analyses without adjustment for background infection prevalence (differences in HR ranging from 0~0.02), except for small differences in the effect of age (shown in **Supplementary Fig. 5**) and region (shown in **Fig. 2**). **(B)-(E) Heterogeneity tests comparing estimated declines between most recent identified infection being with the previous/penultimate vs earlier variant.** Two-sided z-test was used to test the significance.

(A) Sensitivity analyses without adjustment for background prevalence	Reinfection in Omicron BA.1 wave (27 December 2021 to 6 February 2022)				Reinfection in Omicron BA.2 wave (14 March 2022 to 22 May 2022)				Reinfection in Omicron BA.4/5 wave (27 June 2022 to 6 November 2022)				Reinfection in Omicron BQ.1/CH.1.1/XBB.1.5 subvariants wave (7 November 2022 to 31 January 2023)			
	HR	95%CI		p-value	HR	95%CI		p-value	HR	95%CI		p-value	HR	95%CI		p-value
**Sex (Male vs Female)**	0.91	0.83	0.99	**0.03**	0.85	0.79	0.91	**1.1e-05**	0.87	0.83	0.90	**5.0e-12**	0.91	0.88	0.95	**5.3e-06**
**Ethnicity (Non-white vs White)**	0.92	0.80	1.06	0.2	0.70	0.61	0.81	**1.9e-06**	0.88	0.82	0.95	**0.002**	0.83	0.76	0.90	**4.6e-06**
**Healthcare worker (Yes vs No)**	1.26	1.07	1.47	**0.005**	1.10	0.95	1.28	0.2	1.05	0.96	1.13	0.3	1.12	1.04	1.22	**0.005**
**Long-term health condition (Yes vs No)**	0.95	0.85	1.07	0.3	1.02	0.94	1.11	0.6	1.06	1.01	1.11	**0.02**	1.03	0.98	1.08	0.2
**Deprivation decile**	0.98	0.97	1.00	**0.04**	1.01	1.00	1.03	0.06	1.00	0.99	1.00	0.4	1.00	0.99	1.00	0.2
**Symptom in most recent previous infection (Yes vs No)**	0.96	0.86	1.08	0.6	0.92	0.84	1.01	0.08	1.04	0.98	1.10	0.2	1.09	1.03	1.14	**0.001**
**Any Ct<30 or LFD positive in previous infections**	1.00	0.79	1.27	0.9	0.81	0.67	0.97	**0.03**	1.01	0.92	1.12	0.7	1.08	0.98	1.18	0.1
**Region (Northern Ireland vs England)**	0.61	0.41	0.89	**0.01**	0.51	0.36	0.72	**9.1e-05**	0.93	0.81	1.07	0.3	1.34	1.19	1.50	**6.9e-07**
**Region (Scotland vs England)**	0.60	0.45	0.78	**1.2e-04**	0.80	0.67	0.96	**0.02**	0.97	0.88	1.06	0.5	0.95	0.87	1.04	0.2
**Region (Wales vs England)**	0.35	0.22	0.56	**1.0e-05**	0.88	0.71	1.08	0.2	1.01	0.90	1.13	0.8	1.18	1.07	1.29	**8.5e-04**
**Number of previous infections (2 vs 1)**	0.94	0.64	1.38	0.7	1.05	0.82	1.34	0.7	1.02	0.94	1.11	0.6	1.01	0.94	1.09	0.7
**Number of previous infections (3 vs 1)**									1.16	0.70	1.93	0.6	1.31	0.95	1.81	**0.1**
**Per 60 days after a most recent Pre-Alpha infection**	1.00	0.89	1.13	0.9	0.98	0.87	1.11	0.7	0.93	0.79	1.10	0.4	0.88	0.63	1.22	0.4
**Per 60 days after a most recent Alpha infection**	0.96	0.86	1.07	0.4	1.00	0.90	1.11	0.9	0.91	0.84	0.98	**0.009**	0.86	0.76	0.97	**0.01**
**Per 60 days after a most recent Delta infection**	0.75	0.61	0.91	**0.004**	1.14	1.07	1.21	**2.2e-05**	1.00	0.97	1.04	0.9	0.89	0.85	0.93	**3.9e-07**
**Per 60 days after a most recent Omicron BA.1 infection**									1.18	1.12	1.23		0.96	0.91	1.01	0.1
**Per 60 days after a most recent Omicron BA.2 infection**									1.31	1.19	1.45		1.18	1.12	1.24	**7.6e-11**
**Per 60 days after a most recent Omicron BA.4/5 infection**													1.44	1.28	1.63	**4.2e-09**

(B) Sensitivity analyses counting participants as being 'at risk' from the date of their first negative test	Omicron BA.1 wave (27 December 2021 to 6 February 2022)				Omicron BA.2 wave (14 March 2022 to 22 May 2022)				Omicron BA.4/5 wave (27 June 2022 to 6 November 2022)				Omicron BQ.1/CH.1.1/XBB.1.5 subvariants wave (7 November 2022 to 31 January 2023)			
	HR	95%CI		p-value	HR	95%CI		p-value	HR	95%CI		p-value	HR	95%CI		p-value
**Sex (Male vs Female)**	0.89	0.81	0.96	**0.005**	0.84	0.78	0.90	**5.0e-08**	0.86	0.83	0.90	**1.2e-13**	0.91	0.88	0.94	**9.1e-06**
**Ethnicity (Non-white vs White)**	0.93	0.81	1.07	0.3	0.71	0.63	0.81	**3.0e-07**	0.88	0.82	0.95	**0.001**	0.84	0.77	0.90	**5.0e-06**
**Healthcare worker (Yes vs No)**	1.25	1.07	1.46	**0.004**	1.16	1.02	1.32	**0.02**	1.04	0.96	1.13	0.4	1.13	1.04	1.22	**0.003**
**Long-term health condition (Yes vs No)**	0.95	0.85	1.06	0.3	1.04	0.96	1.12	0.3	1.08	1.03	1.13	**0.002**	1.04	0.99	1.09	0.2
**Deprivation percentile**	0.98	0.96	0.99	**0.004**	1.01	0.99	1.02	0.3	1.00	0.99	1.00	0.3	0.99	0.99	1.00	0.2
**Symptom in most recent previous infection (Yes vs No)**	0.93	0.84	1.04	0.2	0.90	0.82	1.01	0.07	1.02	0.97	1.08	0.4	1.08	1.02	1.15	**0.005**
**Any Ct<30 or LFD positive in all previous infections**	1.00	0.80	1.25	0.9	0.89	0.75	1.04	0.1	1.01	0.91	1.11	0.8	1.09	0.99	1.19	0.07
**Region (Northern Ireland vs England)**	0.54	0.37	0.79	**0.002**	0.62	0.43	0.89	**0.008**	0.95	0.83	1.09	0.4	1.08	0.94	1.21	0.3
**Region (Scotland vs England)**	0.71	0.55	0.93	**0.01**	0.73	0.60	0.88	**0.004**	0.95	0.86	1.04	0.2	0.97	0.91	1.06	0.7
**Region (Wales vs England)**	0.45	0.30	0.69	**1.9e-04**	0.85	0.70	1.05	0.2	0.92	0.83	1.02	0.1	1.02	0.94	1.13	0.2
**Number of previous infections (2 vs 1)**	0.87	0.62	1.24	0.4	0.92	0.76	1.11	0.3	1.02	0.94	1.11	0.6	1.00	0.93	1.07	0.9
**Number of previous infections (3 vs 1)**									1.23	0.77	1.95	0.4	1.19	0.87	1.63	0.3
**Per 60 days after a most recent Pre-Alpha infection**	0.99	0.89	1.12	0.9	0.97	0.86	1.09	0.6	0.92	0.78	1.08	0.3	0.88	0.63	1.23	0.5
**Per 60 days after a most recent Alpha infection**	0.95	0.85	1.06	0.3	0.99	0.89	1.10	0.8	0.89	0.83	0.96	**0.003**	0.86	0.76	0.97	**0.01**
**Per 60 days after a most recent Delta infection**	1.46	1.36	1.58	**1.0e-21**	1.09	1.04	1.15	**6.0e-04**	1.00	0.96	1.03	0.8	0.89	0.85	0.93	**3.5e-07**
**Per 60 days after a most recent Omicron BA.1 infection**					2.30	1.93	2.72	**<2e-16**	1.24	1.19	1.30	**<2e-16**	0.96	0.91	1.02	0.1
**Per 60 days after a most recent Omicron BA.2 infection**									1.75	1.63	1.87	**<2e-16**	1.24	1.18	1.29	**<2e-16**
**Per 60 days after a most recent Omicron BA.4/5 infection**									3.63	2.47	5.32	**3.7e-11**	1.70	1.58	1.85	**<2e-16**
**Prevalence (per 1% higher)**	1.21	1.15	1.27	**6.0e-15**	1.25	1.17	1.30	**2.8e-15**	1.33	1.26	1.40	**<2e-16**	1.30	1.25	1.36	**<2e-16**

(C) Sensitivity analyses only including infections defined by positive test results from the study	Reinfection in Omicron BA.1 wave (27 December 2021 to 6 February 2022)				Reinfection in Omicron BA.2 wave (14 March 2022 to 22 May 2022)				Reinfection in Omicron BA.4/5 wave (27 June 2022 to 6 November 2022)				Reinfection in Omicron BQ.1/CH.1.1/XBB.1.5 subvariants wave (7 November 2022 to 31 January 2023)			
	HR	95%CI		p-value	HR	95%CI		p-value	HR	95%CI		p-value	HR	95%CI		p-value
**Sex (Male vs Female)**	0.95	0.76	1.19	0.6	0.87	0.75	1.00	0.05	0.87	0.81	0.93	**1.5e-04**	0.93	0.87	0.99	**0.02**
**Ethnicity (Non-white vs White)**	1.17	0.80	1.70	0.4	0.92	0.69	1.22	0.6	0.95	0.82	1.10	0.5	0.89	0.77	1.01	0.08
**Healthcare worker (Yes vs No)**	0.97	0.59	1.59	0.9	0.91	0.64	1.29	0.6	0.91	0.77	1.09	0.3	1.07	0.93	1.24	0.3
**Long-term health condition (Yes vs No)**	0.66	0.49	0.89	**0.007**	0.92	0.77	1.09	0.3	1.00	0.91	1.09	0.9	1.01	0.93	1.08	0.9
**Deprivation decile**	0.98	0.94	1.01	0.3	1.02	0.99	1.05	0.2	1.00	0.99	1.01	0.9	1.00	0.98	1.01	0.5
**Symptom in most recent previous infection (Yes vs No)**	0.92	0.70	1.19	0.7	0.91	0.80	1.11	0.5	1.02	0.92	1.10	0.8	1.07	0.98	1.16	0.1
**Any Ct<30 or LFD positive in previous infections**	1.09	0.63	1.90	0.8	0.74	0.51	1.07	0.1	0.97	0.82	1.15	0.7	1.04	0.89	1.22	0.6
**Region (Northern Ireland vs England)**	1.96	1.30	2.97	**0.001**	0.95	0.67	1.36	0.8	1.28	1.11	1.48	**9.4e-04**	1.58	1.40	1.78	**1.1e-13**
**Region (Scotland vs England)**	1.86	1.36	2.56	**1.1e-04**	1.45	1.18	1.78	**4.6e-04**	1.33	1.20	1.47	**6.8e-08**	1.12	1.02	1.22	**0.02**
**Region (Wales vs England)**	1.11	0.68	1.82	0.7	1.61	1.28	2.03	**5.1e-05**	1.39	1.23	156	**5.8e-08**	1.38	1.25	1.53	**4.1e-10**
**Number of previous infections (2 vs 1)**	0.67	0.16	2.76	0.6	0.94	0.48	1.82	0.7	1.14	0.94	1.39	0.07	1.15	1.00	1.32	0.06
**Number of previous infections (3 vs 1)**									2.27	0.94	5.46	0.6	2.16	1.30	3.60	**0.003**
**Per 60 days after a most recent Pre-Alpha infection**	0.95	0.71	1.26	0.7	0.93	0.76	1.16	0.5	1.07	0.81	1.41	0.6	0.58	0.36	0.93	**0.02**
**Per 60 days after a most recent Alpha infection**	0.91	0.70	1.18	0.5	0.92	0.77	1.11	0.4	0.89	0.79	1.01	0.06	0.86	0.72	1.04	0.1
**Per 60 days after a most recent Delta infection**	0.88	0.53	1.47	0.6	1.19	1.04	1.36	**0.01**	1.01	0.93	1.09	0.9	0.89	0.82	0.98	**0.01**
**Per 60 days after a most recent Omicron BA.1 infection**									1.16	1.06	1.27	**0.001**	0.90	0.82	0.98	**0.02**
**Per 60 days after a most recent Omicron BA.2 infection**									1.29	1.11	1.49	**7.6e-04**	1.18	1.10	1.27	**1.9e-06**
**Per 60 days after a most recent Omicron BA.4/5 infection**													1.42	1.22	1.66	**7.0e-06**
**Prevalence (per 1% higher)**	1.19	1.13	1.25	**2.8e-12**	1.23	1.16	1.30	**4.8e-13**	1.32	1.26	1.39	**<2e-16**	1.31	1.25	1.37	**<2e-16**

Supplementary Table 4. Sensitivity analyses estimating adjusted hazard ratios (HRs) with 95% CIs in parametric survival models examining the risk of reinfection in multiple Omicron infection waves (Omicron BA.1, BA.2, BA.4/5, and BQ.1/CH.1.1/XBB.1.5). Two-sided z-test was used to test the significance of model coefficients. The 95% CIs are calculated as the exponent of the estimates ± 1.96 × standard error of the estimates. Table A shows the results in models without adjustment for background infection prevalence. Table B shows the results in models counting participants as being 'at risk' from the date of their first negative PCR test in the study following each infection rather than 120 days after their previous infection. Results remain broadly similar to the primary analysis. Table C shows the results in models only including infections defined by positive test results from the study (so not influenced by test seeking behaviour).

	Study worker home visits (before 31 July 2022) N=467,376	Remote data collection (vast majority after 11 July 2022*) N=319,996
**Age**		
**Median**	54	56
**IQR**	39, 67	42, 68
**Sex**		
**Female**	250233 (54%)	174292 (54%)
**Male**	217143 (46%)	145704 (46%)
**Ethnicity**		
**Non-white**	32811 (7%)	20612 (6%)
**White**	434565 (93%)	299384 (94%)
**Reporting working in healthcare**		
**No**	446558 (96%)	304659 (95%)
**Yes**	20818 (4%)	15337 (5%)
**Reporting having a long-term health condition**		
**No**	342908 (73%)	235145 (73%)
**Yes**	124468 (27%)	4851 (27%)

* remote data collection started for the vast majority of participants from 11 July 2022; a very small number (~4,000) participated in a pilot rollout phase from May 2022.

Supplementary Table 5. Characteristics of participants who were visited by study workers before 31 July 2022 and who participated in remote data collection.

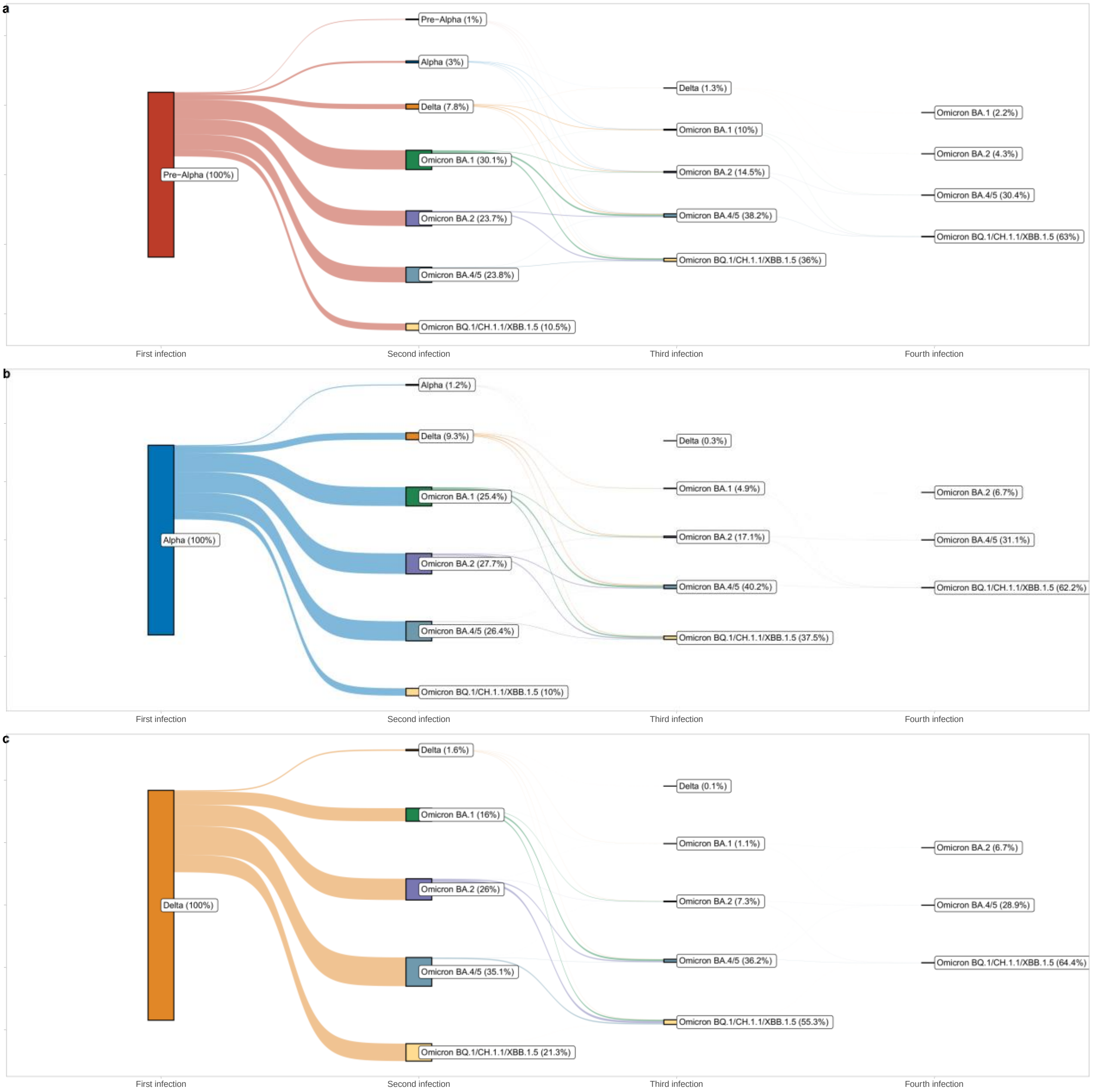

Supplementary Fig. 1. Percentages of with first, second, third, and fourth infection by variants of the first infection (Pre-Alpha, Alpha, and Delta) and subsequent infections.

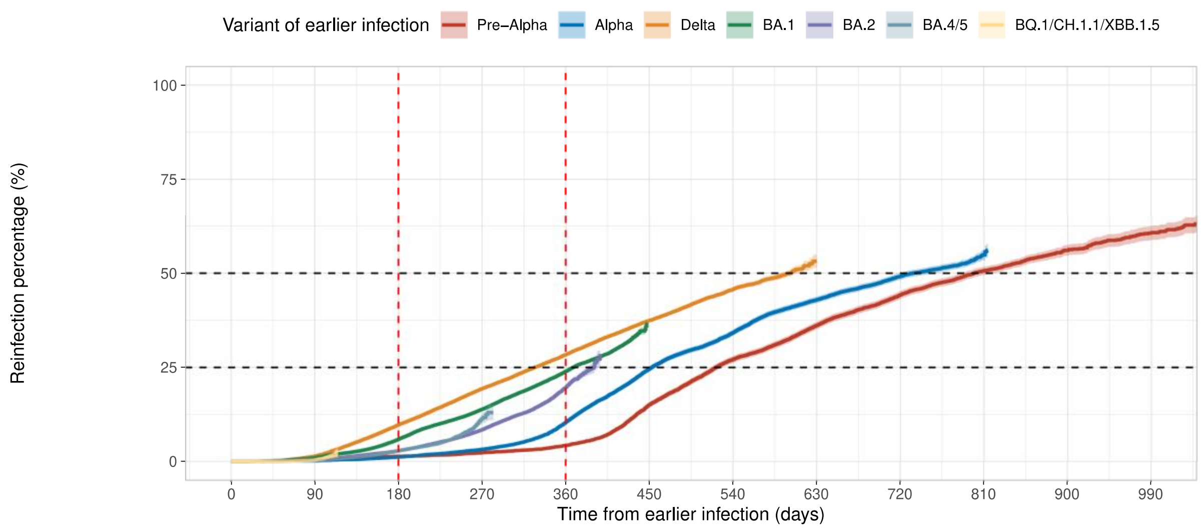

Number at risk

Variant of earlier infection	0	90	180	270	360	450	540	630	720	810	900	990
Pre–Alpha	11448	10648	10090	9446	8911	7678	6207	4954	4123	2805	417	280
Alpha	15888	14855	14003	13156	11811	9487	7416	5999	4673	168	0	0
Delta	36808	34777	30527	24942	20543	14773	4492	102	0	0	0	0
BA.1	53209	49922	42181	36720	28448	56	0	0	0	0	0	0
BA.2	68369	61804	55931	47837	3223	0	0	0	0	0	0	0
BA.4/5	66474	63392	36693	369	0	0	0	0	0	0	0	0
BQ.1/CH.1.1/XBB.1.5	34573	2782	0	0	0	0	0	0	0	0	0	0

Time from earlier infection (days)

Cumulative number of events

Variant of earlier infection	0	90	180	270	360	450	540	630	720	810	900	990
Pre–Alpha	0	54	151	246	430	1416	2480	3206	3817	4260	4404	4443
Alpha	0	25	183	463	1426	3254	4402	5344	5966	6203	6207	6207
Delta	0	518	3400	6508	9183	11672	12936	13148	13152	13152	13152	13152
BA.1	0	570	2869	6319	10453	12199	12202	12202	12202	12202	12202	12202
BA.2	0	269	1728	4850	7971	8066	8066	8066	8066	8066	8066	8066
BA.4/5	0	165	1462	2349	2355	2355	2355	2355	2355	2355	2355	2355
BQ.1/CH.1.1/XBB.1.5	0	37	56	56	56	56	56	56	56	56	56	56

Time from earlier infection (days)

Supplementary Fig. 2. Unadjusted percentage reinfected over time from the earlier infection by variant of earlier infection using Kaplan-Meier estimation. Survival models were built from the start of the earlier infection and censored at each participant's last visit date. Black horizontal dashed lines show the time to 25% and 50% of the subgroup becoming reinfected. Red vertical dashed lines show the percentage reinfected 6 and 12 months after the earlier infection. Number at risk and cumulative number of events are shown in tables by 90 days. 95% CIs are calculated as estimates ± 1.96 × standard error of the estimates.

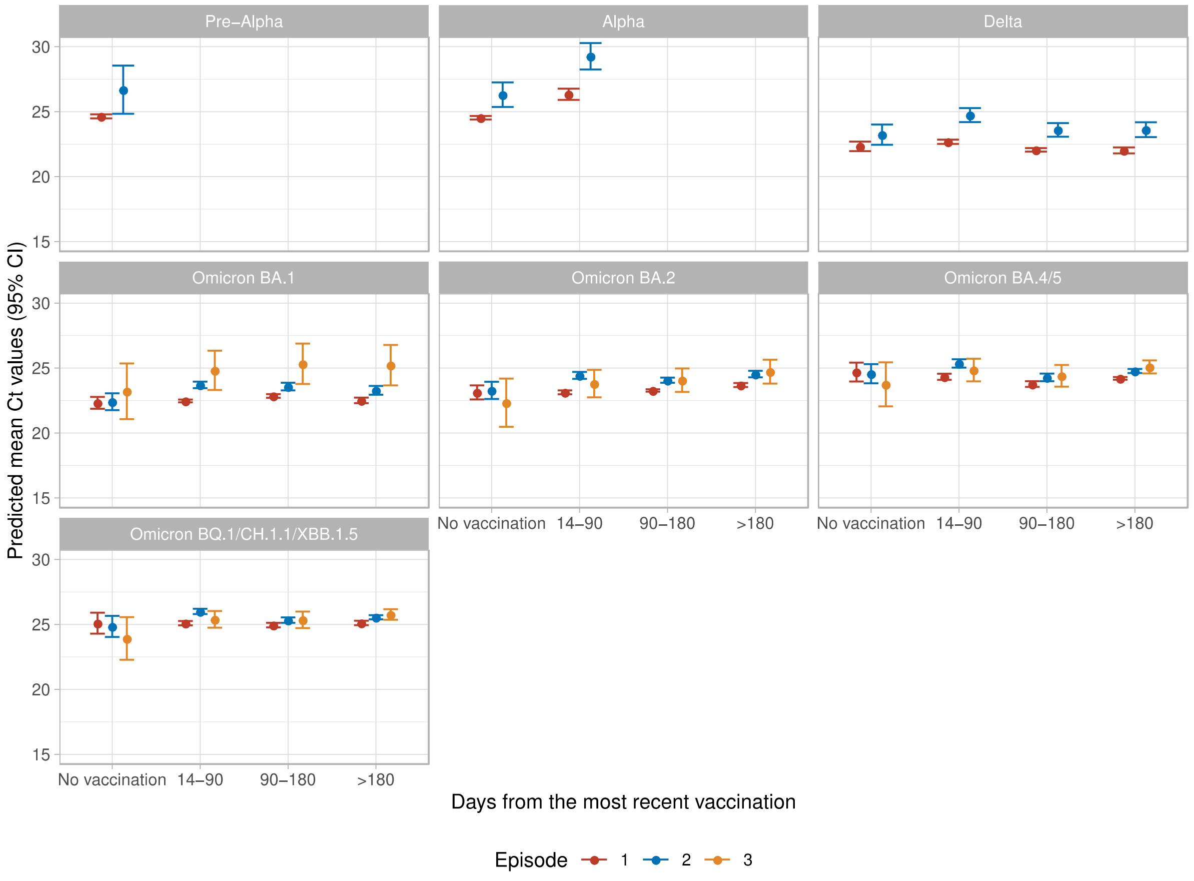

Supplementary Fig. 3. Predicted mean Ct values (95% CIs) by infection variant, days from the most recent vaccination, and infection (first, second, or third), using infections with Ct measured in CIS only (i.e. excluding Ct values from the same assay but measured through national testing programmes). n=105,040 infections were included in the model. The 95% CIs are calculated as predictions ± 1.96 × standard error of the predictions. Predictions are plotted at the reference value of other variables (age=40 years, female, white ethnicity, not reporting working in healthcare, not reporting having a long-term health condition, deprivation percentile=60, reporting symptoms, having one positive test in the infection.

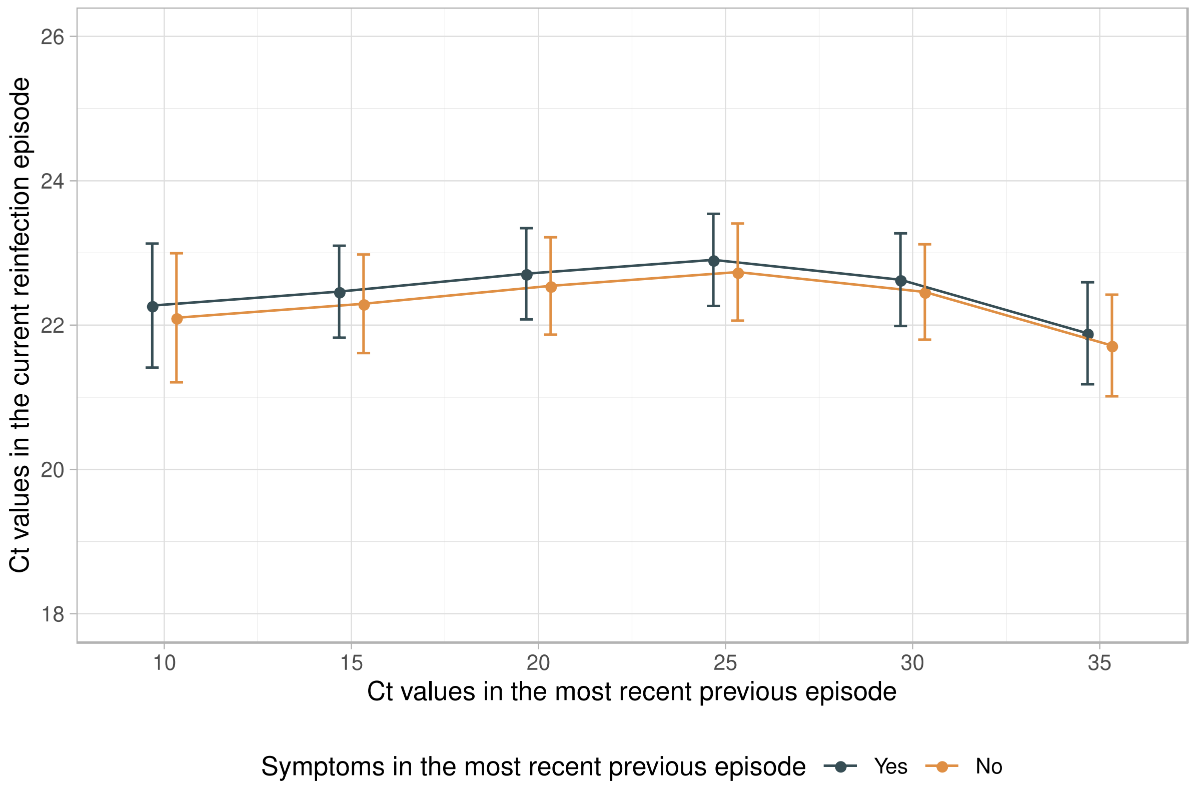

Supplementary Fig. 4. Association between mean Ct values in the current reinfection with Ct values and symptoms in the most recent previous infection. The 95% CIs are calculated as estimates ± 1.96 × standard error of the estimates. Adjusted (**Supplementary Table 2**) for age, sex, ethnicity, reporting working in healthcare, reporting having a long-term health condition, deprivation percentile, reported symptoms in this infection, number of positive tests in the infection, infection variant, time from most recent vaccination.

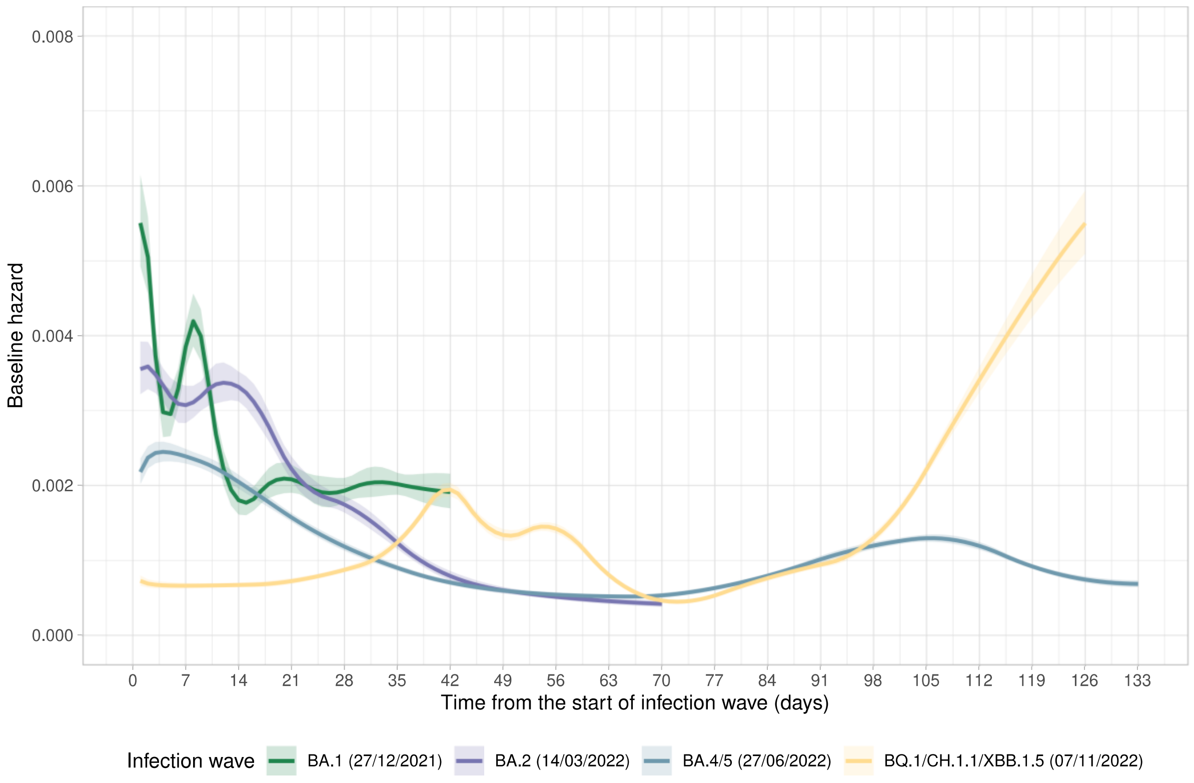

Supplementary Fig. 5. Baseline reinfection risk over calendar time from the start of the BA.1, BA.2, BA.4/5, and BQ.1/CH.1.1/XBB.1.5 infection waves estimated using unadjusted flexible parametric survival models. 95% CIs are calculated as mean estimates ± 1.96 × standard error of the estimates. n=42,582, 83,382, 164,263, and 184,566 adults who were at risk of BA.1, BA.2, BA.4/5, and BQ.1/CH.1.1/XBB.1.5 reinfections were included in the models, respectively.

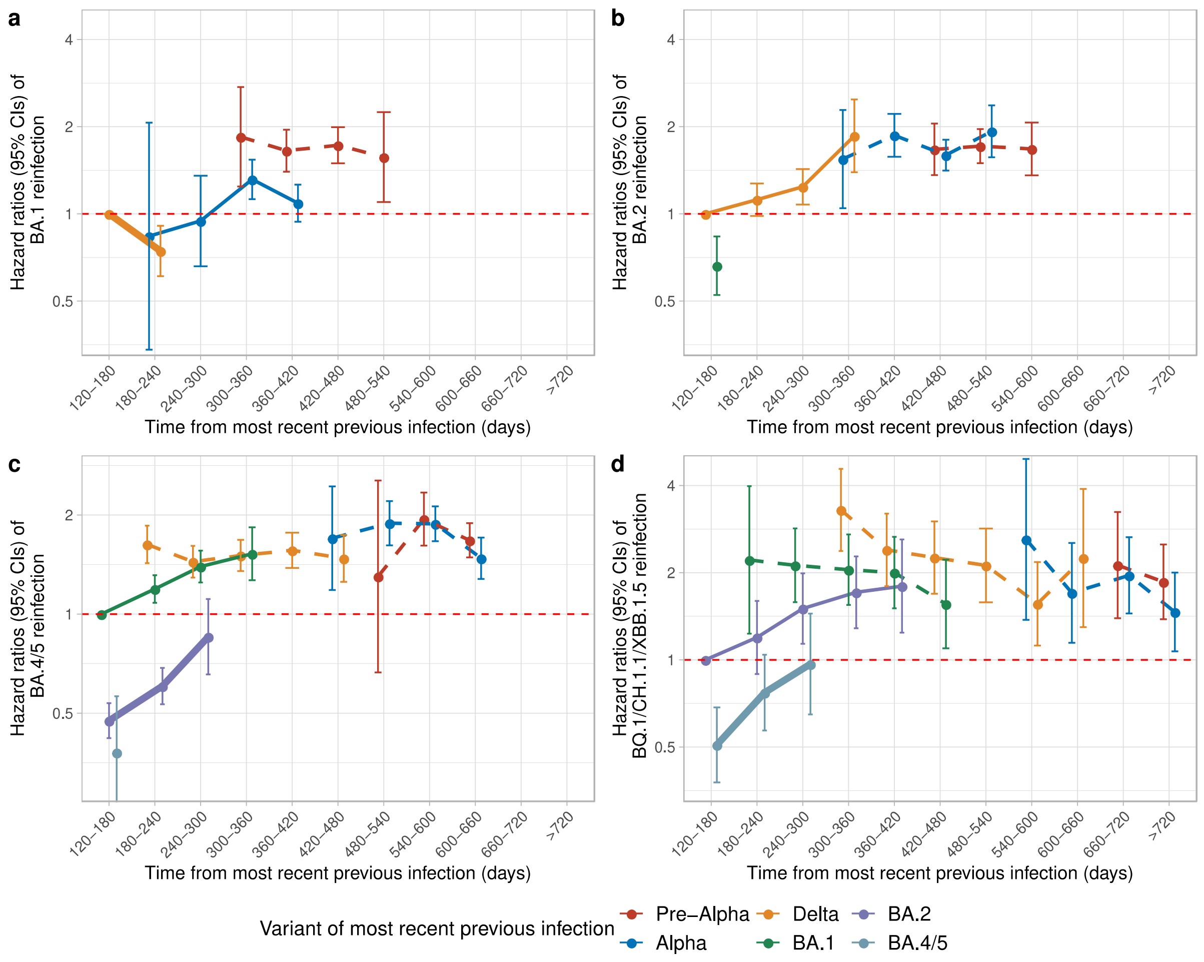

Supplementary Fig. 6. Risk (mean hazard ratios with 95% CIs) of reinfections during Omicron BA.1 (a), Omicron BA.2 (b), Omicron BA.4/5 (c) and Omicron BQ.1/CH.1.1/XBB.1.5 (d) waves by time from most recent previous infection and variant of most recent previous infection. n=42,582, 83,382, 164,263, and 184,566 adults who were at risk of BA.1, BA.2, BA.4/5, and BQ.1/CH.1.1/XBB.1.5 reinfections were included in the models, respectively. Time from previous infection was a time-updated covariate categorised as 120-180, 180-240, 240-300, 300-360, 360-420, 420-480, 480-540, 540-600, 600-660, 660-720, >720 days and its effect modelled categorically (**Fig. 2** shows results modelling the categories as a linear trend). Risk is presented versus a reference category of 120-180 days from an infection in the wave starting ~6 months before the current wave (Delta for BA.1 and BA.2 waves, BA.1 for BA.4/5 waves and BA.2 for BQ.1/CH.1.1/XBB.1.5 waves). Line type and width represent the sequence of variants for better comparisons across waves (thick solid line represents the previous variant, thin solid line represents the penultimate variant, and dashed lines represent earlier variants). The 95% CIs are calculated as the exponent of estimates ± 1.96 × standard error of the estimates. Adjusted for time-fixed covariates age, sex, ethnicity, reporting working in healthcare, reporting having a long-term health condition, deprivation percentile, infection variant, region, number of previous infections, symptoms in most recent infection and whether any previous infection had Ct<30 or was LFD positive; and time-updated time from most recent vaccination. Results remain similar in sensitivity analyses without adjustment for background infection prevalence.

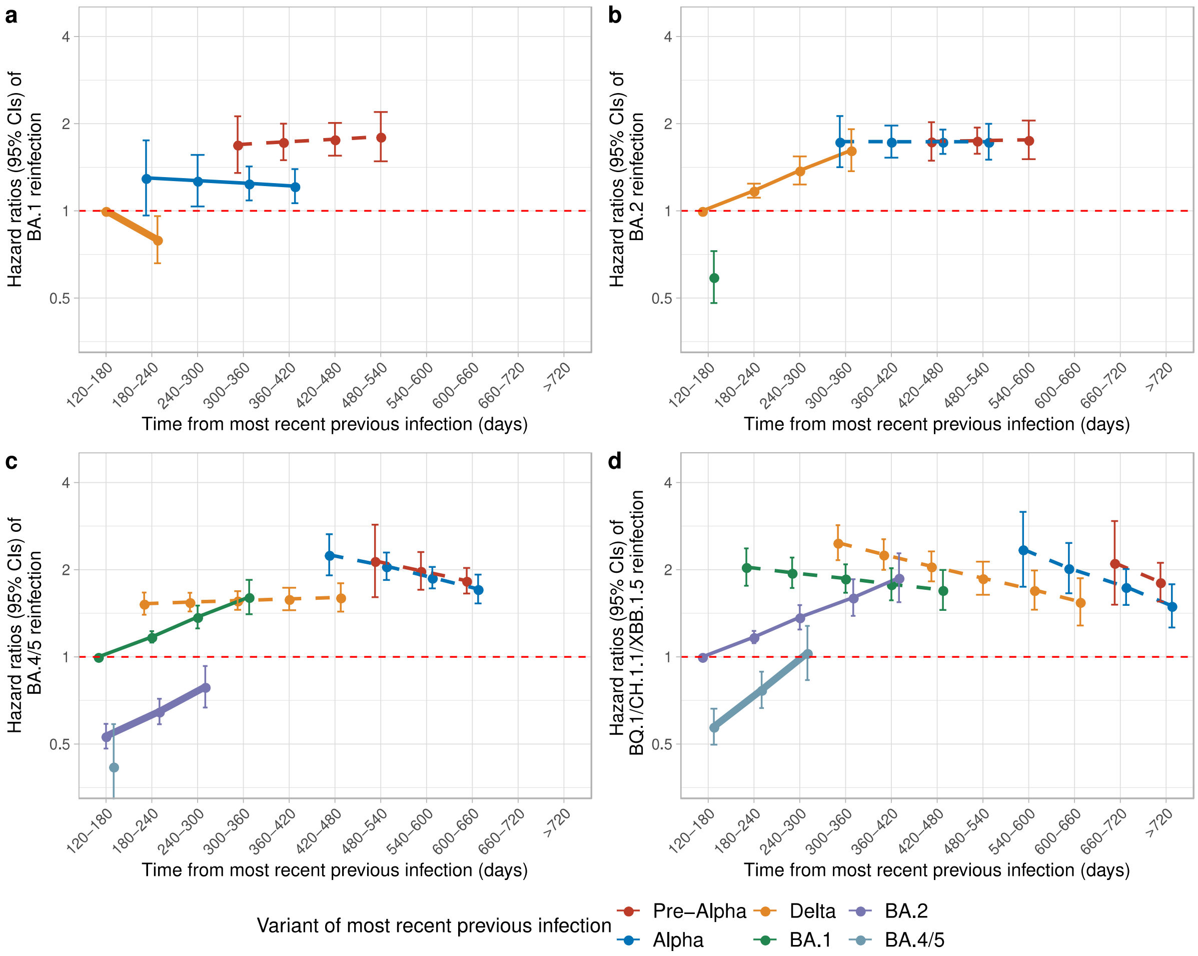

Supplementary Fig. 7. Risk (mean hazard ratios with 95% CIs) of reinfections during the Omicron BA.1 (a), Omicron BA.2 (b), Omicron BA.4/5 (c) and Omicron BQ.1/CH.1.1/XBB.1.5 (d) waves by time from most recent previous infection and variant of most recent previous infection in sensitivity analysis further including whether participants 'thought they had had COVID-19' to potentially reduce missed infections. n=42,582, 83,382, 164,263, and 184,566 adults who were at risk of BA.1, BA.2, BA.4/5, and BQ.1/CH.1.1/XBB.1.5 reinfections were included in the models, respectively. Time from previous infection was a time-updated covariate categorised as 0-60, 60-120, 120-180, 180-240, 240-300, 300-360, 360-420, 420-480, 480-540, 540-600, 600-660, 660-720, >720 days and its effect modelled as a trend over these categories. Risk is presented versus a reference category of 120-180 days from an infection in the wave starting ~6 months before the current wave (Delta for BA.1 and BA.2 waves, BA.1 for BA.4/5 waves and BA.2 for BQ.1/CH.1.1/XBB.1.5 waves). Line type and width represent the sequence of variants for better comparisons across waves (thick solid line represents the previous variant, thin solid line represents the penultimate variant, and dashed lines represent earlier variants). The 95% CIs are calculated as exponent of estimates ± 1.96 × standard error of the estimates. Adjusted for time-fixed covariates age, sex, ethnicity, reporting working in healthcare, reporting having a long-term health condition, deprivation percentile, infection variant, region, number of previous infections, symptoms in most recent infection and whether any previous infection had Ct<30 or was LFD positive; and time-updated time from most recent vaccination.

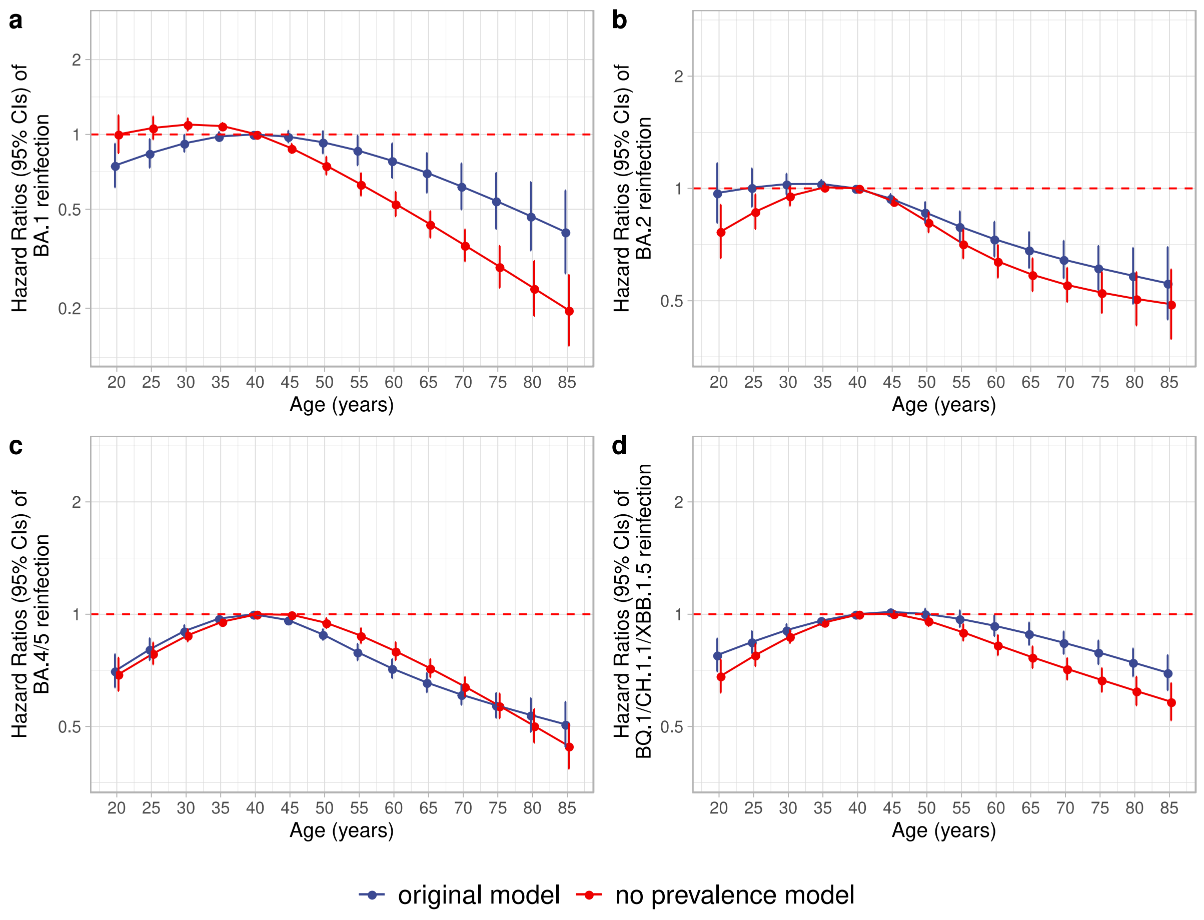

Supplementary Fig. 8. Independent association between age (mean hazard ratios with 95% CIs) and risk of reinfection in the Omicron BA.1, BA.2, BA.4/5, and BQ.1/CH.1.1/XBB.1.5 waves. n=42,582, 83,382, 164,263, and 184,566 adults who were at risk of BA.1, BA.2, BA.4/5, and BQ.1/CH.1.1/XBB.1.5 reinfections were included in the models, respectively. The 95% CIs are calculated as the exponent of estimates ± 1.96 × standard error of the estimates. The differences in hazard ratios by age between the model with and without adjustment for background infection prevalence generally show slight attenuation towards the null adjusting for background prevalence. See **Supplementary Tables 3, 4** for other factors.

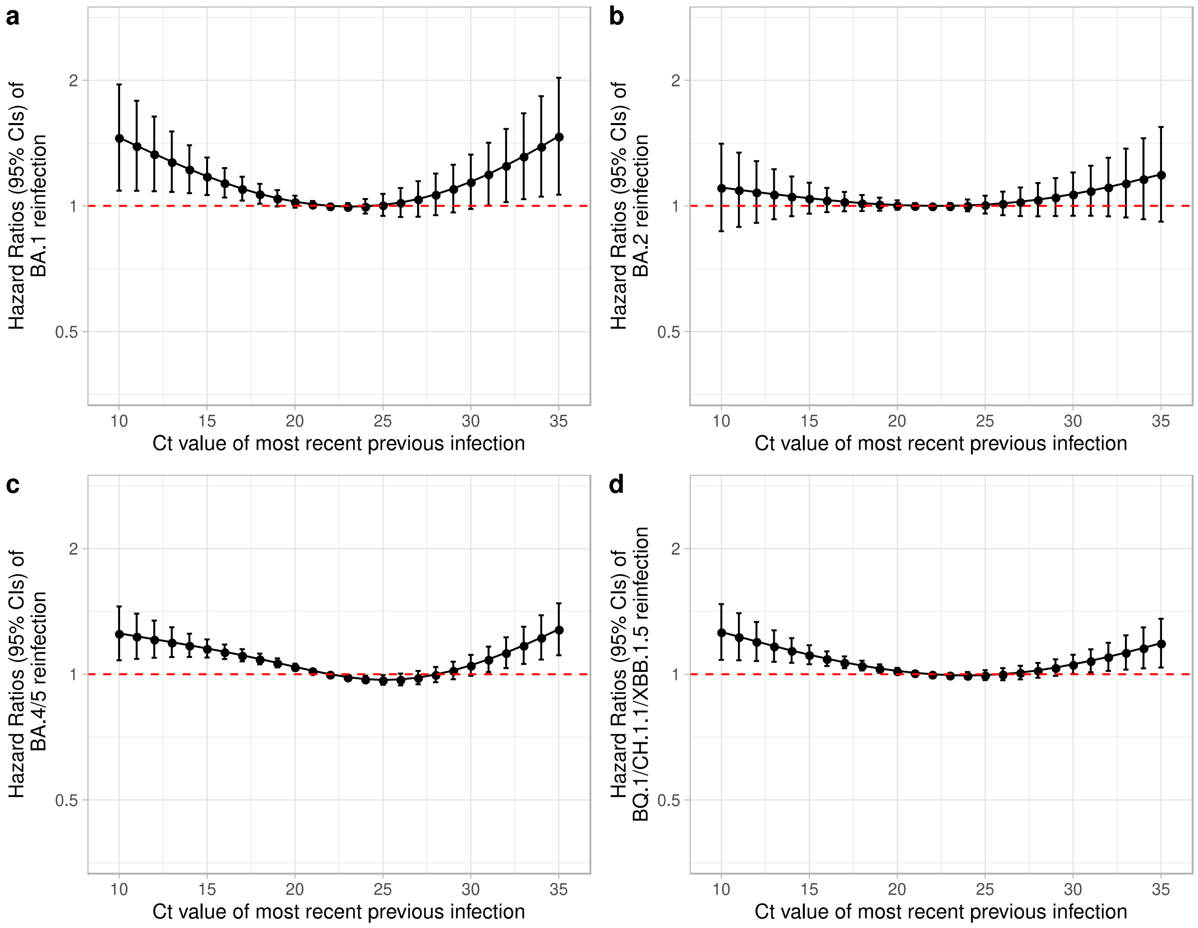

Supplementary Fig. 9. Independent association between Ct value of the previous infection and risk (mean hazard ratios with 95% CIs) of reinfection in the Omicron BA.1, BA.2, BA.4/5, and BQ.1/CH.1.1/XBB.1.5 waves. n=42,582, 83,382, 164,263, and 184,566 adults who were at risk of BA.1, BA.2, BA.4/5, and BQ.1/CH.1.1/XBB.1.5 reinfections were included in the models, respectively. The 95% CIs are calculated as the exponent of estimates ± 1.96 × standard error of the estimates. Results remain unchanged in sensitivity analyses without adjustment for background infection prevalence. See **Supplementary Tables 3, 4** for other factors.

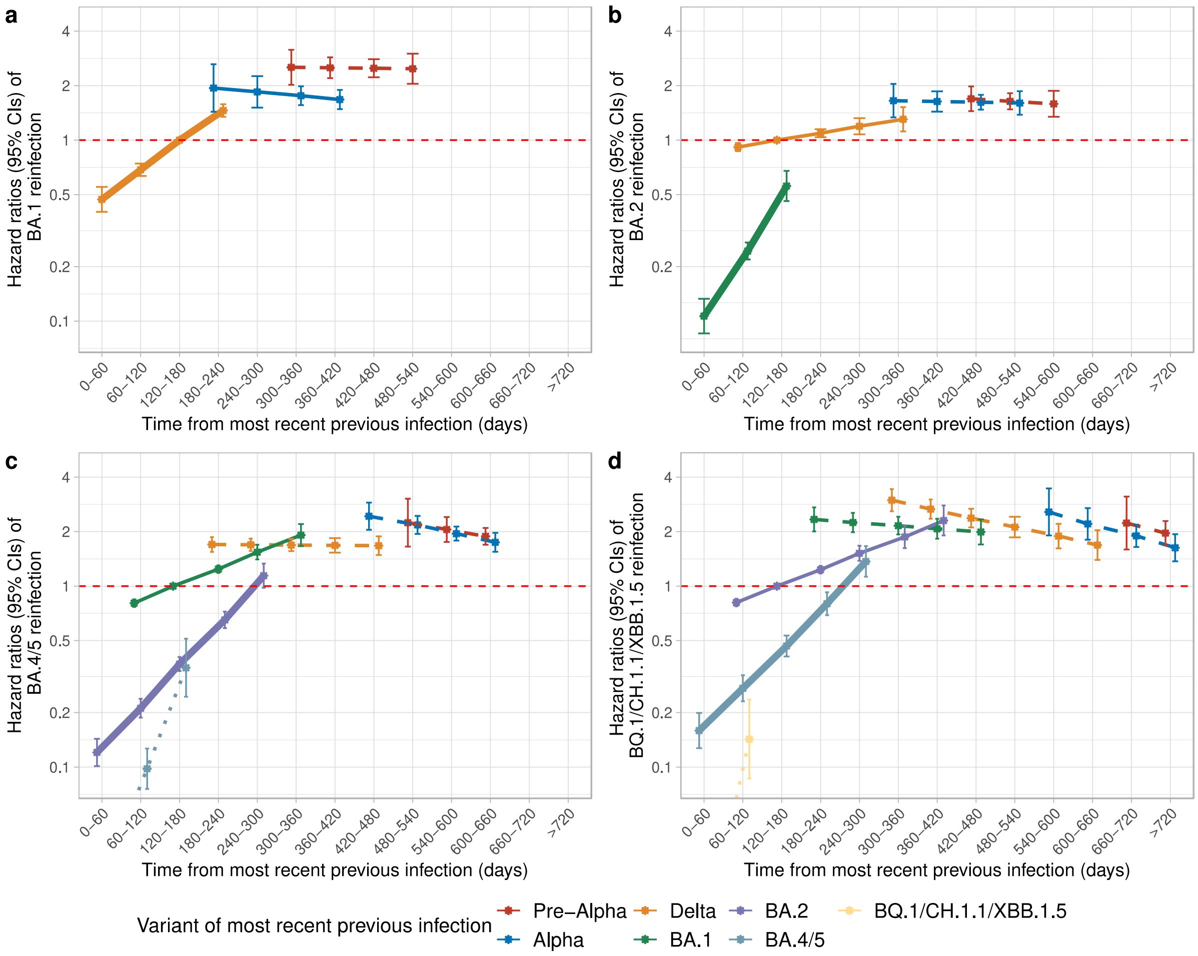

Supplementary Fig. 10. Risk (mean hazard ratios with 95% CIs) of reinfections during the Omicron BA.1 (a), Omicron BA.2 (b), Omicron BA.4/5 (c) and Omicron BQ.1/CH.1.1/XBB.1.5 (d) waves by time from most recent previous infection and variant of most recent previous infection in sensitivity analysis counting participants as being 'at risk' from the date of their first negative study PCR test following the infection. n=75,267, 143,759, 194,335, and 210,612 adults who were at risk of BA.1, BA.2, BA.4/5, and BQ.1/CH.1.1/XBB.1.5 reinfections were included in the models, respectively. Time from previous infection was a time-updated covariate categorised as 0-60, 60-120, 120-180, 180-240, 240-300, 300-360, 360-420, 420-480, 480-540, 540-600, 600-660, 660-720, >720 days and its effect modelled as a trend over these categories (see **Supplementary Fig. 11** for categorical effects). Risk is presented versus a reference category of 120-180 days from an infection in the wave starting ~6 months before the current wave (Delta for BA.1 and BA.2 waves, BA.1 for BA.4/5 waves and BA.2 for BQ.1/CH.1.1/XBB.1.5 waves). Line type and width represent the sequence of variants for better comparisons across waves (thick solid line represents the previous variant, thin solid line represents the penultimate variant, and dashed lines represent earlier variants). The 95% CIs are calculated as exponent of estimates ± 1.96 × standard error of the estimates. Adjusted for time-fixed covariates age, sex, ethnicity, reporting working in healthcare, reporting having a long-term health condition, deprivation percentile, infection variant, region, number of previous infections, symptoms in most recent infection and whether any previous infection had Ct<30 or was LFD positive; and time-updated time from most recent vaccination.

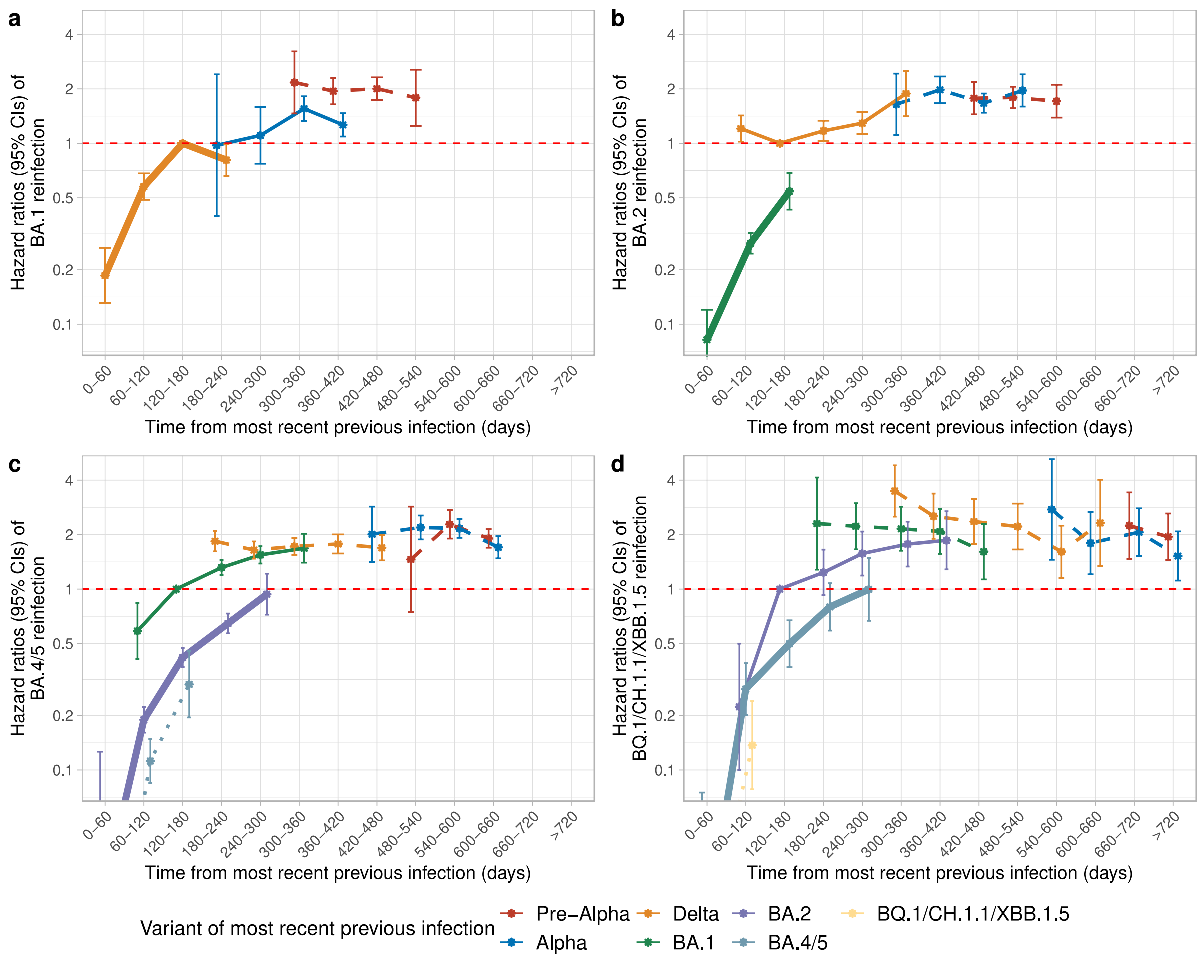

Supplementary Fig. 11. Risk (mean hazard ratios with 95% CIs) of reinfections during Omicron BA.1 (a), Omicron BA.2 (b), Omicron BA.4/5 (c) and Omicron BQ.1/CH.1.1/XBB.1.5 (d) waves by time from most recent previous infection and variant of most recent previous infection in sensitivity analyses counting participants as being 'at risk' from the date of their first negative study PCR test following the infection. n=75,267, 143,759, 194,335, and 210,612 adults who were at risk of BA.1, BA.2, BA.4/5, and BQ.1/CH.1.1/XBB.1.5 reinfections were included in the models, respectively. Time from previous infection was a time-updated covariate categorised as 0-60, 60-120, 120-180, 180-240, 240-300, 300-360, 360-420, 420-480, 480-540, 540-600, 600-660, 660-720, >720 days and its effect modelled categorically (**Supplementary Fig. 10** shows results modelling the categories as a linear trend). Risk is presented versus a reference category of 120-180 days from an infection in the wave starting ~6 months before the current wave (Delta for BA.1 and BA.2 waves, BA.1 for BA.4/5 waves and BA.2 for BQ.1/CH.1.1/XBB.1.5 waves). Line type and width represent the sequence of variants for better comparisons across waves (thick solid line represents the previous variant, thin solid line represents the penultimate variant, and dashed lines represent earlier variants). The 95% CIs are calculated as the exponent of estimates ± 1.96 × standard error of the estimates. Adjusted for time-fixed covariates age, sex, ethnicity, reporting working in healthcare, reporting having a long-term health condition, deprivation percentile, infection variant, region, number of previous infections, symptoms in most recent infection and whether any previous infection had Ct<30 or was LFD positive; and time-updated time from most recent vaccination.

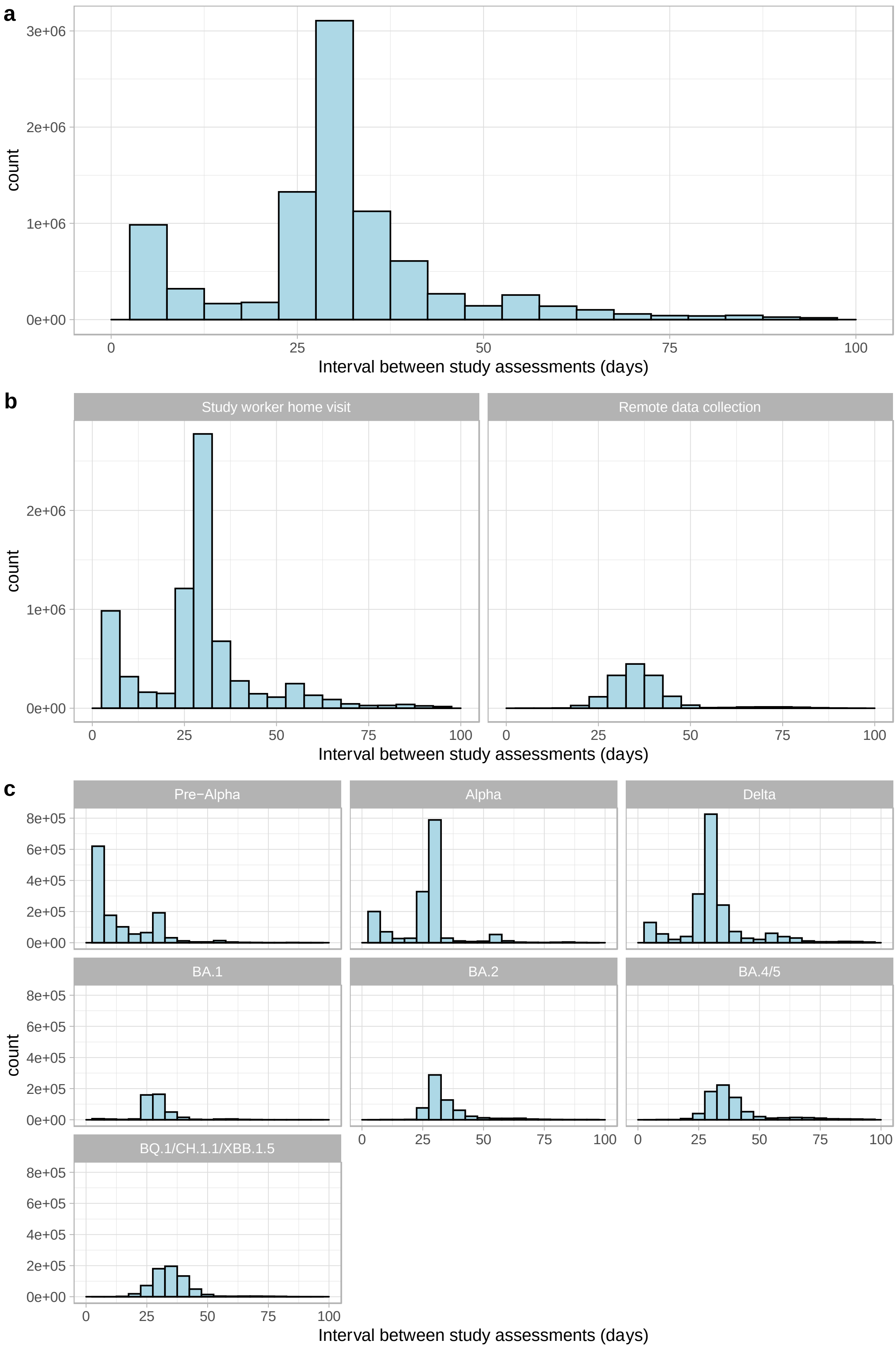
a
count
3e+06
2e+06
1e+06
0e+00
0
25
50
75
100
Interval between study assessments (days)
b
Study worker home visit
Remote data collection
count
2e+06
1e+06
0e+00
Interval between study assessments (days)
c
Pre−Alpha
Alpha
Delta
BA.1
BA.2
BA.4/5
BQ.1/CH.1.1/XBB.1.5
8e+05
6e+05
4e+05
2e+05
0e+00
Interval between study assessments (days)

Supplementary Fig. 12. Distribution of the intervals between survey assessments included in the study. Assessments were planned on a 28–42-day cycle to achieve overall targets for swabs taken per month**.** Most intervals between assessments are <45 days, reducing the number of missed infections due to missed assessments (panel a). Additional information about positive tests taken outside the study was also used to classify previous infection status, see Methods. After 11 July 2022 the survey moved to remote data collection (questionnaires completed online or by telephone) with participants returning test kits by post or courier. The median interval being assessments was 35 days (IQR 31-40), indicating that participants still performed and posted the tests on schedule (panel b) (note that immediately after enrolment assessments were weekly for the first month, corresponding to the early part of the distribution for study worker visits in panel b only, since no new participants were recruited after January 2022). Distribution of intervals between survey assessments were similar across different waves (except Pre-Alpha which had more of the early weekly assessments by design) (panel c). The median (IQR) intervals were 8 (7-24), 28 (25-28), 29 (27-34), 28 (26-31), 31 (28-36), 36 (31-41), 34 (29-39) days for Pre-Alpha, Alpha, Delta, BA.1, BA.2, BA.5, BQ.1/CH.1.1/XBB.1.5 waves, respectively.
