# Supplementary material for: Risk of SARS-CoV-2 reinfection during multiple Omicron variant waves in the UK general population
Source: Nat Commun. 2024 Feb 2;15:1008. doi: 10.1038/s41467-024-44973-1 (PMC10837445; doi:10.1038/s41467-024-44973-1)
Supplement: Supplementary file 3 — Reporting Summary [file 41467_2024_44973_MOESM3_ESM.pdf]

nature portfolio

Corresponding author(s): Jia Wei

Last updated by author(s): Dec 26, 2023

Reporting Summary

Nature Portfolio wishes to improve the reproducibility of the work that we publish. This form provides structure for consistency and transparency in reporting. For further information on Nature Portfolio policies, see our Editorial Policies and the Editorial Policy Checklist.

Statistics

For all statistical analyses, confirm that the following items are present in the figure legend, table legend, main text, or Methods section.

n/a	Confirmed	
☐	☒	The exact sample size (*n*) for each experimental group/condition, given as a discrete number and unit of measurement
☐	☒	A statement on whether measurements were taken from distinct samples or whether the same sample was measured repeatedly
☐	☒	The statistical test(s) used AND whether they are one- or two-sided<br>*Only common tests should be described solely by name; describe more complex techniques in the Methods section.*
☐	☒	A description of all covariates tested
☐	☒	A description of any assumptions or corrections, such as tests of normality and adjustment for multiple comparisons
☐	☒	A full description of the statistical parameters including central tendency (e.g. means) or other basic estimates (e.g. regression coefficient) AND variation (e.g. standard deviation) or associated estimates of uncertainty (e.g. confidence intervals)
☐	☒	For null hypothesis testing, the test statistic (e.g. *F*, *t*, *r*) with confidence intervals, effect sizes, degrees of freedom and *P* value noted<br>*Give P values as exact values whenever suitable.*
☒	☐	For Bayesian analysis, information on the choice of priors and Markov chain Monte Carlo settings
☒	☐	For hierarchical and complex designs, identification of the appropriate level for tests and full reporting of outcomes
☒	☐	Estimates of effect sizes (e.g. Cohen's *d*, Pearson's *r*), indicating how they were calculated

Our web collection on statistics for biologists contains articles on many of the points above.

Software and code

Policy information about availability of computer code

Data collection	De-identified study data were accessed through the Office for National Statistics (ONS) Secure Research Service (SRS). The data available in SRS were prepared and processed using Stata MP 17.
Data analysis	PCR outputs were analysed using UgenTec FastFinder 3.300.5, with an assay-specific algorithm and decision mechanism that allows conversion of amplification assay raw data into test results with minimal manual intervention.<br>Analyses were performed in R 4.0 using the following packages: tidyverse (version 1.3.1), ggsankey (version 0.0), MASS (version 7.3-53), nnet (version 7.3-12), arsenal (version 3.4.0), gmodels (version 2.18.1), ggeffects(version 0.14.3), cowplot (version 1.1.1), emmeans (version 1.5.1), survival (version 3.2-7), survminer(version 0.4.8), and rstpm2 (version 1.5.2).<br>A copy of the analysis code is available at https://github.com/jiaweioxford/COVID19_reinfection.

For manuscripts utilizing custom algorithms or software that are central to the research but not yet described in published literature, software must be made available to editors and reviewers. We strongly encourage code deposition in a community repository (e.g. GitHub). See the Nature Portfolio guidelines for submitting code & software for further information.

Data

Policy information about availability of data

All manuscripts must include a data availability statement. This statement should provide the following information, where applicable:

- Accession codes, unique identifiers, or web links for publicly available datasets
- A description of any restrictions on data availability
- For clinical datasets or third party data, please ensure that the statement adheres to our policy

De-identified study data (including the COVID-19 Infection Survey data and vaccination data from NIMS) are available for access by accredited researchers in the ONS Secure Research Service (SRS) for accredited research purposes under part 5, chapter 5 of the Digital Economy Act 2017. Individuals can apply to be an accredited researcher using the short form on https://researchaccreditationservice.ons.gov.uk/ons/ONS_registration.ofml. Accreditation requires completion of a short free course on accessing the SRS. To request access to data in the SRS, researchers must submit a research project application for accreditation in the Research Accreditation Service (RAS). Research project applications are considered by the project team and the Research Accreditation Panel (RAP) established by the UK Statistics Authority at regular meetings. Project application example guidance and an exemplar of a research project application are available. A complete record of accredited researchers and their projects is published on the UK Statistics Authority website to ensure transparency of access to research data. For further information about accreditation, contact Research.Support@ons.gov.uk or visit the SRS website.

Research involving human participants, their data, or biological material

Policy information about studies with human participants or human data. See also policy information about sex, gender (identity/presentation), and sexual orientation and race, ethnicity and racism.

Reporting on sex and gender	Participants from private households were randomly selected for enrollment, and sex was based on self-reporting. A total of 134,353 females and 111,542 males were included in the analysis. Models were adjusted for sex.
Reporting on race, ethnicity, or other socially relevant groupings	Participants from private households were randomly selected for enrollment, and ethnicity was based on self-reporting. We grouped all non-White ethnicity due to small numbers. A total of 229,566 reported White ethnicity, and 16,329 reported non-White ethnicity. Models were adjusted for ethnicity.
Population characteristics	We included 245,895 participants over18y. The median age was 53y (Interquartile range [IQR] 40-65), 134,353 (54.6%) participants were female, and 229,566 (93.4%) reported white ethnicity. 60,627 (24.7%) reported having a long-term health condition, and 13,123 (5.3%) were healthcare workers.
Recruitment	The Office for National Statistics (ONS) COVID-19 Infection Survey (CIS) is a large household survey with longitudinal follow-up (ISRCTN21086382, https://www.ndm.ox.ac.uk/covid-19/covid-19-infection-survey/protocol-and-information-sheets) (details in20). The study received ethical approval from the South Central Berkshire B Research Ethics Committee (20/SC/0195). Private households are randomly selected on a continuous basis from address lists and previous surveys to provide a representative sample across the UK. Following verbal agreement to participate, a study worker visited each selected household to take written informed consent for individuals aged 2 years and over. Parents or carers provided consent for those aged 2-15 years; those aged 10-15 years also provided written assent. To reduce the probability that invited individuals/households decide not to respond to the survey they are paid generously to participate in the survey, see https://www.ons.gov.uk/surveys/informationforhouseholdsandindividuals/householdandindividualsurveys/covid19infectionsurveycis/howtotakepart. All participants who completed the enrolment visit was offered a £50 voucher, and one £25 voucher for each further visit. For the current analysis we only included individuals aged 16 years and over.<br><br>Nevertheless a certain degree of non-response is inevitable. While certain factors might drive non-response to invitations to participate, adjustment for covariates that may influence selection into the sample ensures that estimates of relative effects are not biased by factors that both influence selection into the sample and the risk of the outcome (model-based inference). Factors that were included in the models are listed below. We cannot exclude the possibility that other unmeasured factors that could influence self-selection into the survey and are not strongly associated with factors already included in the model could bias the result.
Ethics oversight	The study received ethical approval from the South Central Berkshire B Research Ethics Committee (20/SC/0195).

Note that full information on the approval of the study protocol must also be provided in the manuscript.

Field-specific reporting

Please select the one below that is the best fit for your research. If you are not sure, read the appropriate sections before making your selection.

☒ Life sciences ☐ Behavioural & social sciences ☐ Ecological, evolutionary & environmental sciences

For a reference copy of the document with all sections, see nature.com/documents/nr-reporting-summary-flat.pdf

Life sciences study design

All studies must disclose on these points even when the disclosure is negative.

Sample size	245,895 participants over 18y who were infected with SARS-CoV-2 based on a positive swab test in the study, national testing programmes or self-reported at any time up to their final study assessment were included. All available data were used for the current study, with the timing of the analysis determined by the duration of follow up available, rather than sample size given the number of participants in the study.
Data exclusions	No available data were excluded from the study.
Replication	All measurements and analytical assays were undertaken once given the scale of the study and cost limitations with 100,000s of assays performed. Serially collected samples from the same participants included demonstrate reproducibility over time.<br>The statistical analyses have been successfully replicated by two individuals.
Randomization	Recruitment randomised - we used data from the UK's Office for National Statistics (ONS) COVID-19 Infection Survey (CIS) (ISRCTN21086382) which randomly selects private households on a continuous basis from address lists and previous surveys conducted by the ONS or the Northern Ireland Statistics and Research Agency to provide a representative sample across the four countries comprising the UK (England, Wales, Northern Ireland, Scotland). No intervention.
Blinding	Not done. This was an observational study with no interventions. Results were returned to participants to support their involvement, but this would not be expected to impact the study findings.

Reporting for specific materials, systems and methods

We require information from authors about some types of materials, experimental systems and methods used in many studies. Here, indicate whether each material, system or method listed is relevant to your study. If you are not sure if a list item applies to your research, read the appropriate section before selecting a response.

Materials & experimental systems

n/a	Involved in the study	
☒	☐	Antibodies
☒	☐	Eukaryotic cell lines
☒	☐	Palaeontology and archaeology
☒	☐	Animals and other organisms
☐	☒	Clinical data
☒	☐	Dual use research of concern
☒	☐	Plants

Methods

n/a	Involved in the study	
☒	☐	ChIP-seq
☒	☐	Flow cytometry
☒	☐	MRI-based neuroimaging

Clinical data

Policy information about clinical studies

All manuscripts should comply with the ICMJE guidelines for publication of clinical research and a completed CONSORT checklist must be included with all submissions.

Clinical trial registration	ISRCTN21086382
Study protocol	https://www.ndm.ox.ac.uk/covid-19/covid-19-infection-survey/protocol-and-information-sheets
Data collection	We used data from the UK's Office for National Statistics (ONS) COVID-19 Infection Survey (CIS) from 28 February 2020 to 13 March 2023. The UK's Office for National Statistics (ONS) COVID-19 Infection Survey (CIS) randomly selects private households on a continuous basis from address lists and previous surveys. Individuals were surveyed on their socio-demographic characteristics, behaviours, and vaccination status. Combined nose and throat swabs were taken from all consenting household members for SARS-CoV-2 PCR testing. For a random 10-20% of households, individuals ≥16 years were invited to provide blood samples monthly for serological testing. Household members of participants who tested positive were also invited to provide blood monthly for follow-up visits.
Outcomes	Our primary research outcomes include:<br>1) Examine the severity of reinfections compared with first infections, using multinomial logistic regression model to assess symptoms, and robust linear regression to assess Ct values in participants who had at least one infection.<br>2) Examine the risk factors for reinfections in multiple Omicron waves of the pandemic using flexible parametric survival models in participants at risk of reinfection.<br><br>Our secondary research outcomes include:<br>1) Examine characteristics of those who had multiple infections through descriptive analyses.<br>2) Examine reinfection percentage using Kaplan-Meier estimation.<br>3) Examine the baseline reinfection risk over calendar time from the start of each infection wave using flexible parametric survival

model.
